# Topics of Public Interest

**Published:** 1913-04

**Authors:** 


### TOPICS OF PUBLIC INTEREST.

PROPOSED EXTENSION OF MEMBERSHIP OF A. M. A. Dr. George H. Simmons published by request, in a recent number of the Jour. of the A. M. A., his plan to include as formal members of the Association, all members of County and State Societies. These aggregate about 70,000, whereas only 36,822 are in formal membership in the A. M. A. To the latter body he would apply the term Fellows. He states very frankly that this is merely a change in name, though having the practical advantage of impressing the affiliation of all members of subsidiary organizations and thus lending moral strength to the national organization. As the management of the Association would remain in the hands of the 150 delegates, the Fellows would lose no prestige by the extension of the membership.

This plan receives our cordial support, subject to the following minor suggestions: If possible, it seems to us that combined membership in county, state and national organizations, should be secured at a flat rate, with division among the respective organizations, according to their financial needs. This is, of course, a point as to whose wisdom only those in close touch with affairs, can decide. It is not one of vital importance and yet there is no question but that professional organization loses much of its potential numeric strength on account of the cost. There are probably 20,000 physicians who contribute nothing toward professional organization at present, but who would pay $5 a year if the benefits of the whole plan of organization could be secured. This would go far toward compensating for the loss of 36,822 additional five dollar fees and the actual expense is comparatively small for additions after the first few thousands.

The second suggestion is by no means original, though radical. It consists merely in restoring and extending the plan of permanent membership so long and so satisfactorily employed by the Medical Society of the State of New York. We believe more and more as opportunities for observation increase, that the greatest obstacle to practically universal support of and participation in professional organization, is the notion that the delegate system prevents the average member from having a voice in the affairs of the organization, especially since the delegates cannot act directly to control details. This belief should not be construed as a personal objection to indirect, representative and executive government of the organized profession. But the old delegate method of securing permanent members, worked well. It limited the actual management of affairs, up to the necessary committees and officers to whose judgment details must always be left, to a body that was large enough to be democratic and still not too unwieldy. Every member of the profession who affiliated himself with the organization had, at once, the practical benefits of the organization, and he also felt that with reasonable restriction as to maturity and manifestation of an active interest, he could take part in the control of professional interests. A very practical disadvantage of the present Houses of Delegates, state and national, is the conflict between strictly medical and official duties. This tends naturally to a government of the profession by men who are not especially interested in the actual practic of medicine but rather in its political aspects. If the delegates were simply representatives of the profession. who could be instructed and if necessary changed, to carry out the wishes of the general membership, and who were directly responsible for the policy of the organization, very little friction would result, but, as a matter of fact, the delegates simply exercise the functions normally devolving upon the entire enfranchised body so that the disaffected argue, with literal truth, that there are "wheels within wheels." Now, to prevent misunderstanding, let us add that we personally care as little about professional politics, in the sense of active participation, as we do about politics of clubs or of the nation; and that we take no stock in the cries of "graft," mismanagement and extinction of liberty; but we do believe that there is a general and proper demand that the organized profession should be kept as near the ideal of a pure democracy as possible.

The plan of affiliated and permanent delegate membership seems to us to conform to this ideal as closely as is practical. It is not a theory but a method which has the precedent of the body which really fathered the A. M. A. It worked well in practice for many years and under conditions which show its applicability, with comparatively minor modifications, to the largest professional concourse which can be expected for the whole country for many years to come. It is only occasionally that the registration of physicians at the A. M. A. meetings is more than four times that at the Medical Society of the State of New York. It is doubtful whether regular and active attendance at the former averages more than double that of the old permanent members of the latter, with allowance for general increase of population. The selection of permanent active members in the national organization would naturally depend on somewhat more rigid requirements and might well be made on a basis of a smaller ratio to aggregate membership. And, as intimated, the actual control of affairs would still remain with officers and committees, the only practical difference being a more direct and immediate check upon them by the general membership. This check, we believe, would do more to relieve a feeling of apprehension and hostility to professional organization than to effect radical changes for, as stated, we do not believe that there are any great abuses requiring correction. On the other hand, the system of permanent membership would prevent the former temporary swaying of the A. M. A. by self appointed political leaders, acting on a large and unselected popular gathering of physicians.

---

INVESTIGATION OF THE WHITE SLAVE TRAFFIC. John D. Rockefeller, Jr., like many other young millionaires, is interested in this study, but along different lines from those often pursued.

---

CATS SUSPECTED OF CONVEYING SMALL POX. Berkley, Cal., is waging a war of extermination in this belief.

**Source of English Words**

| | |
|---|---|
| Anglo Saxon and English | 3681 |
| Dutch | 207 |
| Low German | 126 |
| German | 333 |
| Scandinavian | 693 |
| Through French— | |
| German | 85 |
| Old High German | 154 |
| Middle High German | 27 |
| Teutonic | 225 |
| Romance Languages | 297 |
| Latin | 4842 |
| Late Latin | 828 |
| Italian | 162 |
| Celtic | 170 |
| Latin (direct) | 2880 |
| Prevencal from Latin | 25 |
| Italian | 99 |
| Spanish | 108 |
| Portugese | 21 |
| Greek, direct or through Latin, Late Latin, French, etc. | 2493 |
| Slavonic | 31 |
| Litheanian | 1 |
| Asiatic (Aryan) | 163 |
| European (non-Aryan) | 20 |
| Semitic—Hebrew | 99 |
| —Arabic | 272 |
| Asiatic, non-Aryan and non-Semitic | 135 |
| African | 32 |
| American | 102 |
| Hybrid | 675 |
| Unknown | 12 |
| Total list traced | 19,160 |

(Literary Digest, January 25, 1913, authority not stated.)

COLLECTION OF CANCER STATISTICS. Dr. H. R. Gaylord announces that the N. Y. State Institute for the Study of Malignant Diseases (Gratwick Laboratory) is collecting various details regarding cancer cases, especially for Buffalo, though, with the co-operation of the State Board of Health, for the state generally. Cancer specimens, obtained at necropsy, will be examined and returned with report. Specimens should be placed immediately in 10 per cent. formalin solution and may be forwarded in an ordinary mailing case. Application should be made, in advance for a supply of cards for the proper notation of data required.

STUDENTS IN NEW YORK STATE. With a total population of 9,450,000, estimated from the average rate of increase from the census returns of 1910, the state had an attendance of 1,956,365 pupils in 1912. 1,329,925 were in grammar schools, 180,010 in high schools, 10,838 in normal and training schools, 41,381 in colleges and professional schools. About 19 per cent. of an average population or about 1,800,000 children, lie between the ages of 5 and 15, and about half as many in the next five-year period, or 4/10 as many, 720,000, in the high school period. In other words, about three-quarters of all children of grammar school age are actually in school and about one-quarter receive a high school education, though all do not complete it. This is an honorable but, nevertheless, a heavy burden, and doubtless accounts, to some degree, for the general complaint of hard times. Almost exactly a quarter of an average total population consists of males of twenty-five years and over, and it is this part of the community that bears almost the total burden of taxation. For the public schools alone, this tax was a little more than fifty-nine million dollars, or a trifle over $25 a head, and we may add that it is worth it, but that it must represent just about so much economy along other lines.

---

RELATIVE TYPHOID FEVER MORTALITY. Pittsburgh proper had a death rate of 13.4 per hundred thousand population in 1910; Allegany City, 46.9. The former had filtered water, the latter unfiltered from essentially the same source.

---

CONTRACT PRACTICE. Dr. Richard Cabot cites the University of California as an example of the benefits of contract physicians to cure and prevent disease. 7000 students pay $5.00 a piece a year, which secures the services of a number of well qualified and well paid physicians, who are relieved both of the financial worries of private practitioners and of the embarrassment that the conscientious physician feels with regard to volunteering advice as to prophylaxis and prompt attention to symptoms. (Note—Some years ago we suggested that physicians might well be employed by medical congregations, analogous to churches, their duties being ministrative, social, educational, and in the way of directing philanthropy. While, on account of abuse, the term contract practice is, to the profession, like a red rag to a bull, it should be clearly understood that the essential idea is all right, that a contract by a group of individuals for permanent medical service, is a form of insurance, and that the flagrant wrongs of contract practice are due to unfair means employed by both contracting parties, and, particularly, because the estimate of the value of the insurance is inadequate.)

TUBERCULOUS CATTLE. Fifteen of forty-four cattle owned by S. J. Champlin of Canewango were found to be tuberculous on February 27 and were shipped to Buffalo to be killed. Further investigation will be made. Why not kill and burn tuberculous cattle on the spot?

---

OFFICIAL SYNTHETICS. Three are recognized in the B. P. of 1898, phenacetin, phenazone and sulphonal; nine in the U. S. P. of 1905 and in the Danish Pharmacopoeia of 1907; twenty-eight in the German of 1910.

---

STREET CAR SERVICE. The International R. R. Co. is removing the smoking compartments from its suburban cars. While theoretically disapproving of smoking, for hygiene and aesthetic reasons, we do not believe in compulsory reform. The majority of men and probably half of the patrons of street and interurban cars smoke. Decent and adequate accommodiations should be provided for them, as in European countries. Buffalo is agitated over the near-end cars. Cars of the 5000 type in Buffalo and Chicago have proved satisfactory. The near-end cars do not run smoothly and, despite very pretty diagrams, the air is heavily charged with CO2. H2S. Allyl sulphid, etc. When overcrowded, much time is lost in loading and discharging passengers. In case of an accident they will prove dangerous. There is a rear, emergency door. We have never witnessed a state of over-crowding considered emergent enough to allow the use of this door, and we have heard a report of a conductor who tried to open one to replace a trolley pole on the wire and found it jammed tight. It is generally held that the European rule of prohibiting passengers to board a car after its seating capacity is exhausted—with a customary allowance of a few standing passengers—would not be tolerable in America, but it does not cause so much delay as is usually believed. It seems to us that a fair compromise would be to apply this rule at starting points and in passing car barns and to require reserve cars to be kept on hand at such points; then to allow any number of passengers to be picked up at intermediate stops till the steps and coupling rod are full, as at present. Also, while there is much to be said in favor of a curfew law, we think that the influence of the street car companies in discouraging night travel is exerted unduly. If necessary, put on small cars, but run them at reasonable intervals all night.

---

X-RAY LESION. Dr. J. M. Scott of Kansas City, one of the pioneer Rontgenologists, has had his right hand amputated as a result of the irritation of constant exposure to the rays. He intends, however, to proceed with his work.

Liability of Surgeon for Failing to Find Appendix. A La Porte, Ind., woman is suing for $16,665 damages. We trust that the suit will fail. Humiliating as the confession may be, there is no detail in the wide range of medicine and surgery where the most expert and most conscientious may not fail. We have personally encountered cases where hernia and orchitis, pneumonia and gall stones, gall bladder and appendix inflammation could not be immediately differentiated, and, as an interne, saw a case in which several competent physicians and surgeons overlooked a fracture of the arm for several days, the patient having given no history leading to suspicion of any surgical complication and the correct diagnosis being made only after a dispute as to whether it was the nurse's duty to brush the hair of a ward patient apparently able to care for herself. In the case of the appendix there are ample reasons why prolonged search should not be made.

---

Michigan Mortality Rate. For 1912, 13:1000. Tuberculosis caused almost exactly one death in 17. A quarter of a century ago civilized communities considered a death rate of 20:1000 as normal, and tuberculosis caused about 1 in 10 of all deaths.

---

The Library of the College of Physicians of Philadelphia contains about 98,000 books of all kinds. About 70,000 are bound.

---

Letchworth Village, the state institution for the seggregation of epileptics and feeble minded persons, largely due to the bequest of the Buffalo philanthropist, will soon be begun. The first group of buildings will include four dormitories, attendants' home, dining hall, laundry, power plant and store house.

---

St. Louis Consumption Cure. Dr. Heath makes claims for a vaccine somewhat analogous to that of Friedmann, but not in such sweeping terms.

---

Municipal Diagnosticians. St. Louis had added to its Health Department staff a chief diagnostician and ten assistants, whose work will be confined mainly if not entreily to contagious diseases. It seems to us that St. Louis is setting rather an expensive precedent that such official recognition may still further increase friction with private practitioners, especially as the diagnosis of contagious diseases often present great difficulties leading to disputes.

OBESITY. Justine Masson died November 28, 1912, in an insane asylum in Montreal. She weighed 780 pounds and was considered the heaviest woman in Canada.

W. H. Robin of Burnham, Ia., weighs 437 pounds and is 5 feet 3 inches tall.

Frederick Hoffman died January 27 in Buffalo, weight 425 pounds.

Everett Shaw died October 17, 1912, at North Abington, Mass., weight 400 pounds, aged 40.

---

CENTENNARIANS. Samuel Neuin, a Pennsylvania German, was born in Berks County in December, 1804, was rejected as too old to engage in the Civil War, and died last February, after only two weeks' illness.

Jacob Ross, born in Springfield, N. J., June 1, 1812, died in Dansville, N. Y., March 2, 1913.

William Redmond died recently in Oakhurst, N. J., aged 103.

Miss Elizabeth Woodbridge Thompson, a descendant of Gov. Saltonstall, died at Saratoga Springs, February 26, aged 102.

Mrs. Mary Reese, whose husband died at 96, died at Gordonville, Pa., March 4, aged 101.

Peter Morrison, recently of Albany, N. Y., now of Sacramento, was robbed of his purse in San Francisco March 6. His hundredth birthday occurred March 11.

Pierre Chamel is living in Paris at the age of 105. For half his life he is said to have lived on milk.

Mrs. John Shirk of Morgantown, Pa., was 100 years old March 1. She is in good health.

---

PHYSICIANS IN EUROPE (See page 360, January, 1913, issue for physicians in the U. S.) The following statistics are approximately correct:

| Country. | Population Millions. | Physicians. | Ratio. |
|---|---|---|---|
| Europe | 437 | 200,000 | 1:2,180 |
| England | 36 | 48,000 | 1:750 |
| France | 40 | 32,000 | 1:1,250 |
| Germany | 65 | 26,000 | 1:2,500 |
| Italy | 35 | 24,000 | 1:1,450 |
| Russia | 160 | 20,000 | 1:8,000 |
| Austria | 28 | 13,000 | 1:2,150 |
| Belgium | 7 | 12,000 | 1:600 |
| Spain | 20 | 8,000 | 1:2,500 |

Injustice to Senator Gallinger. In the March issue, in alluding to the two physicians in the U. S. Senate, we unintentionally did Dr. Gallinger an injustice, though we followed what appeared to be good authority and merely stated that he was supposed to be opposed to vaccination and animal experimentation, following our custom in eliminating any comment of an offensive nature, as we always concede the right of anyone to hold any opinion, however opposed to our own. One of our editorial acquaintances calls attention to the fact that Dr. Gallinger has represented New Hampshire, not Vermont, in the U. S. Senate for over 25 years and that the Medical Society of the District of Columbia has publicly expressed its appreciation of his efforts on behalf of the profession during his public career in the Senate. We are greatly obliged for the correction and, while we can scarcely make a formal apology without implying that it is derogatory to a man's intelligence to hold an opinion different from that which we represent, we are gratified to note that both of the medical men in the Senate are in accord with the general professional sentiment. Their united influence will be a source of strength to the profession in its efforts to secure public recognition of the necessity of legislation in the interests of the public welfare.

---

Instructor for Miraj Medical School. A teacher of physiology, chemistry, physics, biology and bacteriology is wanted. This school is maintained in connection with the Presbyterian Mission Hospital at Miraj, West India. Nearly 10,000 out patients and over 1700 indoor patients are treated and clinical experience will be afforded, though most of the appointee's time will be taken up with teaching. Traveling expenses, quarters at $50 a month, are the perquisites, with opportunity for permanent service as a medical missionary. The applicant must be a Christian. Address Mr. Wilbert B. Smith, 600 Lexington Ave., New York City.

---

The Kind of Vaccination Argument That Counts. Between Jan. 13 and Feb. 12 every member of the Penny Road School, near Dundee, Ill., who had not been vaccinated, developed smallpox. The cases included the teacher, her mother, and seven children. No child in the school who had been vaccinated nor any member of the families afflicted, similarly protected, contracted the disease. Authority, Chicago Board of Health. Anti-vaccinationists can not be convinced by general statistics of the before-and-after kind, nor by rehashes of fifty-year-old literature, nor by scientific arguments. Up-to-date reports of this kind, subject to investigation, supported by names and dates, with accurate statements of the fate of the unvaccinated and the escape of the vaccinated, will eventually convert all but fanatics.

---

Mortality of Physicians. Various journals, quoting, with and without credit, the mortality statistics of the *Jour. of the A. M. A.*, are, we think, giving too favorable an idea of the longevity of physicians. In 1912, 2,120 physicians are known to have died in the U. S. and Canada. "Reckoning on a conservative estimate of 150,000 physicians," the mortality is 14.13 per thousand, the average for the previous decade having been 15.93. The average mortality for adults of corresponding age, that is 24 or 25 and upward, is about 21 per thousand and, while the medical profession is not a particularly hazardous occupation, and while its members have a theoretic knowledge of hygiene, it does not seem probable that the latter can be practically applied to effect a reduction of approximately a third in the death rate. Polk's Directory, which, if anything, errs in the direction of including men only nominally physicians or of retaining names after the removal of the men, gives a total of slightly less than 136,000 for the U. S. and Canada. On this basis, the mortality is increased to about 15.6. But we do not believe that the full mortality of the profession is, or can be, recorded, even by the most perfect system. We find a considerable number of deaths of retired physicians, especially of those who have left the profession for other lines of activity, quite accidentally, and this convinces us that a good many names escape the statistics altogether. There is a natural tendency for physicians who have retired, either from age or by entering other businesses, to lose touch with the profession, often to remove from their previous locations, where their decease would be noted by their colleagues. In business and social ways one encounters a surprising number of "reformed" physicians.

Ninety physicians died of violence, beside 36 suicides. Ten of the violent deaths resulted from automobiles.

---

Hospitals for Indians. The Committee on Indian Affairs of the United States Senate has approved an Indian appropriation bill providing for hospitals and other means for treating tuberculosis among the Indians. The appropriation amounts to $307,000.

---

Pure Water on Trains. The Treasury Department issued from Washington on January 29 regulations prohibiting the supplying of any but "certified pure" water on trains and steamboats in interstate commerce. The regulations require further that all water containers be scalded at least once a week and that any ice used in cooling the water must also be "certified pure." The requirements of individual drinking cups on common carriers engaged in interstate commerce was made by the Public Health Service some time ago.

For Medical Research. The faculty of medicine of the University of Toronto, Canada, has received an endowment fund for medical research, the income of which will probably amount to $25,000 a year.

---

Legislation Against Fee-Splitting. An amendment to the Medical Practice Act of Missouri has been introduced by Woodward, which has for its purpose a legislative solution of the fee-splitting evil:

"Sec. 8319a. It shall be unlawful for any physician or surgeon to divide or offer to divide his fee or charge for performing any surgical operation with any other physician or physicians, surgeon or surgeons; and any physician or surgeon so offending, on conviction thereof, shall be fined in a sum not to exceed $500, and such conviction shall operate as a revocation of his license to practice medicine and surgery."

---

Course of Clinical Lectures and Demonstrations in the out-patient hall of the New York Skin and Cancer Hospital on the following Wednesday afternoons at 4:15 o'clock on Surgical Diseases of the Skin: April 2, April 9, April 16, April 23, April 30, May 7, Dr. Bulkley. Surgical Treatment of Malignant Diseases: May 14, Dr. Bainbridge. Each lecture will be illustrated by cases, models, colored plates, photographs, etc.

